# Normotensive, Oversized Pheochromocytoma in Twin-Pregnancy: Analysis of Therapeutic Challenges in a Rare Case

**DOI:** 10.1155/2019/7141060

**Published:** 2019-05-22

**Authors:** Bora Özveren, Halim Ulugöl, Levent Türkeri

**Affiliations:** ^1^Acibadem Mehmet Ali Aydinlar University, School of Medicine, Department of Urology, Turkey; ^2^Acibadem Mehmet Ali Aydinlar University, School of Medicine, Department of Anesthesiology & Reanimation, Turkey

## Abstract

An asymptomatic, normotensive 36-year-old woman in the second trimester of a twin-gestation was diagnosed with a 11 cm adrenal pheochromocytoma. Considering the hemodynamic stability of the patient, tumor size, and gestational age, the therapeutic decision of a multidisciplinary team ensued open surgical excision without any preoperative antihypertensive preparation. Following successful removal of pheochromocytoma, the patient had a normal subsequent course of pregnancy and cesarean section delivery of healthy twins at term. This unique case of a normotensive, incidental, large-sized pheochromocytoma in a twin-pregnancy illustrates that the decisions of management in such a rare occurrence should be based on individual features of the patient. Our experience supports that *α*-adrenergic blockade may not be essential in normotensive pheochromocytoma in pregnancy and open-surgery remains as a safe approach in the management of large adrenal tumors in twin-pregnant patients following a multidisciplinary consultation.

## 1. Introduction

Pheochromocytoma (PC) in pregnancy is extremely rare, with an estimated incidence of 1 in 50,000 patients [1]. Historically, PC in pregnancy carried significant risks with mortality rates for both mother and fetus of up to 58% [2]. Maternal and fetal survival greatly depend on a timely diagnosis, an appropriate medical management, and a correct timing of delivery and surgery. When diagnosed antenatally, PC is associated with more successful outcomes, with a maternal mortality rate of 0-2% but still significant risk of fetal loss at a rate of 11-15% [3].

Current report presents the management of a unique case of a twin-pregnancy with an incidentally discovered, asymptomatic large adrenal PC.

## 2. Case Presentation

**Figure 1:**
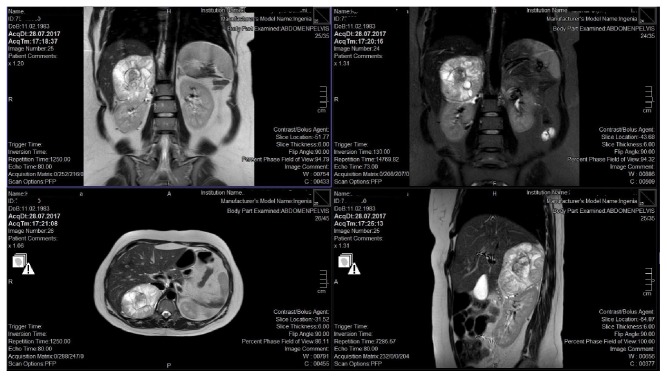
MRI examination showing a 11×7.5 cm right suprarenal hypervascular mass with mixed signal intensity in T2-weighted images.

A 36-year-old woman (G4,P2), at the 15th gestational week of twin-pregnancy following IVF-embryo transfer, was found to have a solid adrenal mass on a regular checkup. MRI revealed a 11×7.5 cm right suprarenal hypervascular mass with mixed signal intensity in T2-weighted images (Figure 1). The diagnosis of PC was confirmed by laboratory analysis (Table 1). The patient had no genetic testing and her family history was not indicative of any hereditary disease. She had two previous vaginal deliveries (14 and 11 years ago) and a history of one abortus at 10-week gestation two years earlier. The patient confirmed that she had no symptoms relating to PC in her previous deliveries. She had no genetic testing and her family history was not indicative of hereditary disease. She was asymptomatic and normotensive and had no hemodynamic instability during pregnancy. She was asymptomatic and normotensive and had no hemodynamic instability during pregnancy. Perinatological ultrasonography revealed normal morphology of dichorionic and diamniotic male and female fetuses.

**Figure 2:**
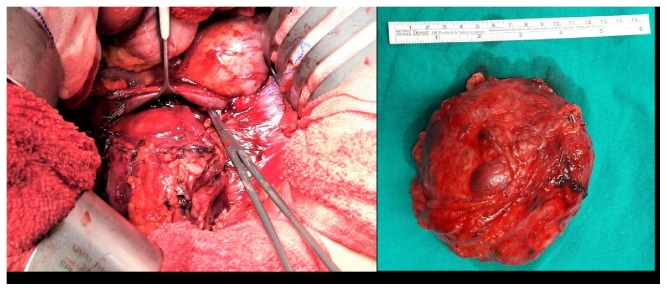
Transperitoneal resection of the hypervascular tumor located above the upper pole of the right kidney, between the vena cava and the lower border of the liver.

A multidisciplinary team consisting of urologists, anesthesiologists, endocrinologist, and obstetricians focused on the therapeutic approach. The patient did not receive any medical treatment for alpha or beta blockade preoperatively. She underwent laparotomy with a subcostal incision and transperitoneal tumor resection at 17 weeks of gestation (Figure 2). During surgery blood pressure (BP) was stabilized with phentolamine and esmolol, with occasional bouts of brief hypertensive periods up to 240 mm Hg systolic pressure. A hypervascular mass with fragile large veins was dissected free of the upper pole of the right kidney, between the vena cava and the lower border of the liver. The estimated blood loss was 1100 ml. The patient was transfused with 3 units of erythrocyte suspensions. The postoperative period was uneventful and she remained hemodynamically stable. Histopathological examinations were in accordance with a PC.

She had a normal subsequent course of pregnancy and cesarean section delivery of healthy twins at term. Nine months after delivery, follow-up ultrasonography revealed no recurrent mass. Urinary and plasma catecholamine levels were in normal range.

## 3. Discussion

Hypertension is the most common presenting feature of pheochromocytoma in pregnancy, reported in 87% [4]. Establishing the diagnosis can be challenging considering the varied modes of presentation, which may mimic other common disorders of pregnancy such as gestational hypertension, atypical preeclampsia, or gestational diabetes.

The medical management primarily aims to prevent hypertensive crisis and usually includes alpha and beta blockers or calcium channel blockers. Surgery is the definitive treatment of PC, but the timing remains a challenging and controversial issue, depending on the gestational age, clinical response to medical treatment, the accessibility of the tumor, and the presence of fetal distress [5].

There are only 2 previous cases of twin-pregnancy complicated by PC. The first patient was a primigravid presenting with high BP, a 7 cm adrenal mass found on ultrasonography, and elevated urinary and plasma catecholamines. Despite an add-on therapy of beta blockers to a high dose of phenoxybenzamine, the pregnancy was complicated with the demise of one of the twins at 29 weeks of gestation and resulting in an emergency cesarean section for the surviving twin shortly after [6]. The second case was a chronic, severely hypertensive woman with 13 weeks of twin-gestation. Evaluation for secondary hypertension disclosed high levels of urinary (nor)metanephrines and a 2.9 cm adrenal mass on MRI. She underwent laparoscopic left adrenalectomy for PC without complications. She had normal BP for the remainder of pregnancy and delivered healthy twins via cesarean section [7].

In addition to being the third report on a twin-gestation with PC in medical literature, the current case has several distinctive features. To the best of our knowledge, we report the largest PC complicating a pregnancy in literature to date, which is treated by open surgical excision in the second trimester with successful maternofetal outcome. Analysis of contemporary series of PC in pregnancy showed that average tumor size is about 3-4 cms (min: 2 cm, max:10 cm) [3, 5, 8, 9]. Adrenal PCs above 7 cm are considered large-sized, and laparoscopic surgery is contraindicated. Moreover, large (5 cm or greater) size is one of the high risk factors predictive of malignant pheochromocytoma [10]. The timing and the method of surgical intervention depend on the size/location of the tumor as well as the gestational age and maternal/fetal response to medical pretreatment. If diagnosed early in pregnancy, it is generally suggested to remove the tumor in the second trimester, following the appropriate medical management of BP and heart rate. In the third trimester, when medical pretreatment is sufficient, it is usually recommended to postpone adrenalectomy until the time of or after delivery. In our case, the considerable size and hypervascularity of the tumor as well as the dimensions of the uterus bearing twin-fetuses substantiated prompt intervention with open surgical approach for safety concerns.

Another foremost difference of our case remains the unusual presentation as an incidental discovery of normotensive PC during twin-pregnancy. The typical clinical manifestation of PC is paroxysmal or sustained hypertension. In the nonpregnant population, absence of hypertension or hemodynamic manifestations are reported in 10 to 40% of incidental PC cases [11]. Despite the frequency of this hemodynamically silent presentation, little is known about the characteristics of normotensive incidentally discovered PCs. Furthermore, there is a paucity of information concerning epidemiology and specific clinical features of normotensive incidental PC in pregnancy due to its extremely rare occurrence. Clinically overt PC in pregnancy may be triggered by several mechanisms such as intra-abdominal pressure, fetal movement, uterine contraction, process of delivery, an abdominal intervention, and general anesthesia. Lafont et al. suggested that normotensive incidental PCs (in nonpregnant patients) are roughly comparable to hypertensive PCs in terms of hemodynamic instability during surgical resection [12]. Furthermore, in their retrospective cohort, they identified no differences in hemodynamic instability in terms of presence or absence of preoperative use of antihypertensive agents.

For nonpregnant patients, the current standard of care consists of a medical pretreatment with *α*-adrenergic receptor blockade for at least 10–14 days before surgery in order to lower the risks of perioperative and postoperative hemodynamic complications. However, in pregnant patients, optimal medical therapy for PC is not clearly defined and target BP is not known, since the published literature largely comprises case reports. While effective control of maternal hypertension is crucial to avoid complications owing to the effects of raised catecholamine levels, medical management requires a balance of maintaining sufficient uteroplacental circulation to prevent fetal growth impairment or demise. The drug of choice, phenoxybenzamine, is a* pregnancy class C* medication that crosses the placenta. Neonates born to mothers receiving phenoxybenzamine should be monitored closely in an intensive care unit, with particular observation for hypotension and respiratory distress [13]. The risk of neonatal cardiac failure may be associated to the large proportion of *α*-receptors in the neonatal heart. However, the relation between phenoxybenzamine and respiratory distress is not clear.

In our case, the hemodynamic stability of the asymptomatic pregnant patient and the probability of adverse effects of antihypertensive medications in the perioperative period warranted a shared-decision of omitting antihypertensive preparation prior to surgery. The current case demonstrates that, in normotensive incidental PC patients, intraoperative hemodynamic stability can successfully be maintained without the requirement of preoperative *α*1-adrenoceptor antagonist therapy.

## 4. Conclusion

We report a patient with 17 weeks of twin-gestation and a diagnosis of asymptomatic normotensive pheochromocytoma who was successfully treated with open-surgery without any preoperative antihypertensive preparation. Multidisciplinary approach and judicious anesthetic management resulted in a successful outcome without compromising the fetal well-being of the twins in this extraordinarily rare case. Considering the lack of treatment guidelines of this rare condition, further evidence is needed to define the best management of pheochromocytoma in pregnant patients.

## Figures and Tables

**Table 1:** Pre- and postoperative catecholamines.

	Preoperative	Postoperative	

*24-hour urinary*			
† Vanillyl mandelic acid	-* *-	6.6 mg/day	(<8.0)
† Metanephrines	699.4 *μ*g/day	78.0 *μ*g/day	(<180)
† Normetanephrines	16510 *μ*g/day	265.9 *μ*g/day	(<419)

*Plasma*			
† Epinephrine	157 pg/ml	8.3 pg/ml	(<85)
† Norepinephrine	9577 pg/ml	108.7 pg/ml	(<420)
† Dopamine	-* *-	3.5 pg/ml	(<50)
